# Internal Fistulas Discovered on Cross-Sectional Imaging Predict Future Intra-Abdominal Abscesses

**DOI:** 10.1016/j.gastha.2025.100684

**Published:** 2025-04-25

**Authors:** Vikas J. Patel, Maher Homsi, Nicholas A. Orriols, Naueen A. Chaudhry, Joseph R. Grajo, Abdullah Malkawi, Patricia Moser, Isaac L. Molina, Robert Case, Fares Ayoub, Nicholas I. Kaufman, Tiffany Lambrou, Ellen M. Zimmermann

**Affiliations:** 1 Division of Gastroenterology, Hepatology and Nutrition, University of Florida College of Medicine, Gainesville, Florida; 2 Division of Abdominal Imaging, Department of Radiology, University of Florida College of Medicine, Gainesville, Florida; 3 Department of Medicine, University of Florida College of Medicine, Gainesville, Florida

**Keywords:** Crohn’s Disease, Fistula, Complications, Cross-Sectional Imaging

## Abstract

**Background and Aims:**

Internal fistulas found on cross-sectional imaging (CSI) performed during routine care of patients with Crohn’s disease (CD) are often considered incidental findings. This study aimed to assess outcomes in patients with internal fistulas on CSI.

**Methods:**

This is a single tertiary care center, retrospective case-control study of CD outcomes. Patients who had magnetic resonance enterography or computer tomography enterography performed between 2007 and 2017 were included. Electronic medical record data up to 2017 were included as variables in logistic regression analysis. CSI was scored by 3 abdominal radiologists blinded to the electronic medical record.

**Results:**

Subjects included 199 CD patients: 63 patients (cases) had internal fistulas on index scan and 136 had no internal fistula. The cases and controls were well-matched for age, race, smoking status, body mass index, and years of disease. During follow-up, cases had a more complicated disease course with higher incidence of intra-abdominal abscess formation (19.1% vs 3.7%; *P* < .001) and abdominal surgery (44.4% vs 24.3%; *P* < .001). Patients with fistula were more likely to require surgery (odds ratio 4.96, *P* < .001) and to develop intra-abdominal abscess (odds ratio 6.05, *P* < .001). The index scan of cases was more likely to demonstrate inflammation (95.2% vs 39.7%; *P* < .001) and stricture (27.0% vs 7.35%; *P* < .001) than controls though the presence of an internal fistula was the only independent variable predictive of intra-abdominal abscess.

**Conclusion:**

CD patients with internal fistulas identified by CSI have worse disease outcomes. Presence of internal fistula is the only independent risk factor for future intra-abdominal abscess regardless of the patient’s symptoms.

## Introduction

Crohn’s disease (CD) is a chronic inflammatory disease with transmural involvement most commonly affecting the distal ileum and colon. Patients with ileal involvement have more aggressive disease and more frequently require surgery, often for penetrating or stricturing complications.^1^, ^2^, ^3^ Cross-sectional imaging including magnetic resonance enterography (MRE) and computer tomography enterography (CTE) are preferred for evaluating the activity of CD in the small intestine and identifying complications including strictures, internal fistulas, obstruction, and abscesses.^4^^,^^5^ Internal fistulas are found on CSI in approximately 15% of the scans performed on CD patients.^6^ When discovered on CSI in the absence of patient symptoms, they are typically considered incidental. Surgeons do not intervene when internal fistulas are discovered incidentally on imaging in otherwise asymptomatic patients.^7^^,^^8^

Internal fistulas are often reported in case series or natural history studies together with more common fistulas such as enterocutaneous or perianal fistulas.^9^^,^^10^ As a result, there are few natural history studies specifically focusing on patients with internal fistulas, and there is a paucity of data to guide optimal management. Here, we performed a retrospective study of CD patients with enteric internal fistulas (ie small-bowel to small-bowel or small-bowel to large-bowel fistulas) found on CSI performed for routine inflammatory bowel disease care. Our hypothesis is that patients with CD who have an internal fistula discovered on CSI will have more subsequent complications and higher health care utilization when compared to control CD patients without an internal fistula. Our study demonstrates that an internal fistula found on routine CSI predicted complicated CD including intra-abdominal abscess formation, regardless of the patient’s symptoms. Our study highlights the importance of internal fistulas in the progression of CD and warrants close observation and aggressive medical therapy.

## Methods

### Study Population

This is a single, tertiary center, retrospective, case-control study. The imaging reports of all patients who underwent abdominal imaging with CTE and MRE at our institution between July 2007 and July 2017 were queried using the Montage radiology search engine (Montage Healthcare Solutions, Philadelphia, PA). To identify scans with an internal fistula, the report database was searched using exam codes pertaining to abdominal cross-sectional imaging for CD (computed tomography or magnetic resonance of the abdomen or pelvis with or without contrast) and the key words: ‘‘fistula or fistulas or enteroenteric fistula”. A control group without a fistula was identified by the key words: “no fistula or no fistulas”. The presence of an internal fistula or no fistula was confirmed by radiographic review (see below). Cases were defined as patients with CD who had an enteroenteric or enterocolic fistula (termed internal fistula for the current study) on CSI. Controls were defined as patients with CD without evidence of internal fistulas by CSI in the same age range. Institutional review board approval to evaluate the electronic medical record (EMR) and retrieve protected health information of patients identified by the Montage search was obtained (IRB201701919).

### Data Collection

EMRs were used to obtain patient information. All patients were diagnosed with CD determined via International Classification of Diseases 9 and International Classification of Diseases 10 codes and confirmed by EMR evidence using standard historical, endoscopic, imaging, and laboratory information. Of the initial 303 patients, 60 were excluded due to lack of follow-up, 29 patients were excluded because they did not have a valid diagnosis of CD, 6 patients did not have complete data available, 7 patients were duplicates, and 2 patients were removed from the fistula group after expert radiology review due to lack of a fistula. One patient had a perianal fistula so was excluded. The study ultimately included 199 unique scans (referred to as the index scan) from 199 unique CD patients (63 cases and 136 controls). EMR data were collected including any history of Crohn’s-related surgery, surgery within 6 months, any history of abscess, or abscess within 1 year of the index scan. In addition, lab work (C-reactive protein, erythrocyte sedimentation rate, hemoglobin, white blood cell [count], albumin) and Harvey Bradshaw Index (HBI) was collected as close as possible to the index scan and greater than half of the collected data were within 1 month. Biologic use was reported as infliximab, adalimumab, vedolizumab, ustekinumab, certolizumab pegol, or natalizumab use within the past 8 weeks. The population characteristics are shown in Table 1.

**Table 1:** Subject Variables Studied by Logistic Regression Modeling

Predictor	Population (n = 199)	Fistula cases (n = 63)	No fistula control (n = 136)	*P* value
Baseline characteristics				
Age (SD)	39.90 (18.06)	39.41 (16.73)	40.14 (18.7)	.79
Gender				
Female	120 (60.30%)	32 (50.79%)	88 (64.71%)	.06
Male	79 (39.70%)	31 (49.21%)	48 (35.29%)	
Race				
Caucasian	162 (81.41%)	47 (74.60%)	115 (84.56%)	.18
African American	30 (15.08%)	12 (19.05%)	18 (13.24%)	
Other	7 (3.52%)	4 (6.35%)	3 (2.21%)	
Smoking status				
Nonsmoker	115 (57.79%)	33 (52.38%)	82 (60.29%)	.36
Current smoker	56 (28.14%)	22 (34.92%)	34 (25.00%)	
Former smoker	28 (14.07%)	8 (12.70%)	20 (14.71%)	
BMI (SD)	24.49 (6.41)	23.62 (6.5)	24.91 (6.35)	.19
History of CD-related surgery prior to index scan	71 (35.67%)	30 (47.62%)	41 (30.15%)	.02
Prior surgery within 6 mo of index scan	9 (4.52%)	5 (7.94%)	4 (2.94%)	.13
History of intra-abdominal abscess	30 (15.07%)	22 (34.92%)	8 (5.88%)	.00
Abscess within 1 y prior to index scan	15 (7.53%)	12 (19.05%)	3 (2.21%)	.00
Average number of years since CD diagnosis (SD)	11.47 (12.00)	12.1 (13.35)	11.17 (11.35)	.61
Index imaging details				
Inflammation	114 (57.28%)	60 (95.24%)	54 (39.71%)	.00
Fixed stricture	27 (13.56%)	17 (26.98%)	10 (7.35%)	.00
Baseline patient information				
HBI baseline (SD)	6.75 (5.11)	6.83 (4.38)	6.72 (5.49)	.90
Abdominal pain	132 (66.33%)	45 (71.43%)	87 (63.97%)	.30
Extraintestinal manifestations	50 (25.12%)	14 (22.22%)	36 (26.47%)	.52
CRP baseline (SD)	25.45 (45.55)	52.05 (66.38)	13.34 (23.9)	.00
ESR baseline (SD)	32.77 (28.40)	43.57 (35.33)	27.59 (22.83)	.00
HGB baseline (SD)	12.11 (2.04)	11.23 (2.07)	12.52 (1.92)	.00
WBC baseline (SD)	8.31 (3.76)	8.45 (3.83)	8.26 (3.75)	.74
Albumin baseline (SD)	3.86 (0.60)	3.62 (0.65)	3.98 (0.56)	.00
Patient follow-up information				
HBI at end of follow-up (SD)	6.29 (4.96)	6.31 (4.77)	6.28 (5.1)	.98
Number of clinic visits (SD)	8.47 (7.74)	9.25 (8.29)	8.11 (7.48)	.33
Number of imaging exams through follow-up (SD)	3.58 (3.64)	4.76 (4.62)	3.04 (2.9)	.00
Number of procedures (SD)	1.44 (1.62)	1.02 (1.22)	1.65 (1.75)	.01
Number of patients with abdominal surgery	51 (25.62%)	28 (44.44%)	33 (24.26%)	.00
Number of patients with ostomy	23 (11.55%)	10 (15.87%)	13 (9.56%)	.21
Number of patients with hospitalizations related to CD (SD)	107 (53.76%)	43 (68.25%)	64 (47.05%)	.01
Number of patients with obstruction	37 (18.59%)	14 (22.22%)	23 (16.91%)	.38
Number of patients with abscess	17 (8.54%)	12 (19.05%)	5 (3.68%)	.00
Number of patients with new manifestations	10 (5.02%)	3 (4.76%)	7 (5.15%)	.91
Number of patients with extraintestinal manifestations	50 (25.12%)	14 (22.22%)	36 (26.47%)	.52
Number of patients with opioid use	63 (31.65%)	22 (34.92%)	41 (30.15%)	.50

BMI, body mass index; CRP, C-reactive protein; ESR, erythrocyte sedimentation rate; HGB, hemoglobin; WBC, white blood cell (count); SD, standard deviation.

### Imaging

A standard MRE protocol was used. MREs were performed on a 1.5 T GE Signa Gemsow (Harvey, IL), 1.5 T Siemens Avanto or 3 T Siemens Verio (Erlangen, Germany). Axial and coronal T2 SS/HASTE (with and without fat saturation sequences) were performed after ingestion of 4 bottles of Volumen and intravenous injection of 1-mg glucagon. Axial and coronal T1 3D FSPGR LAVA/VIBE sequences were obtained before and after intravenous injection of an extracellular gadolinium-based contrast agent. Axial diffusion-weighted images and coronal cine 2-dimensional steady state free precession FIESTA/True FISP (fast imaging with steady state precession) sequences were performed.^11^

CTE imaging studies were conducted using a 64 slice Toshiba NM PET CT 20. CTEs were performed after ingestion of 4 bottles of Volumen and intravenous injection of 150-mL Omnipaque 350 at a rate of 3 mL/sec. Axial 2-mm thin sections were reconstructed with 3-mm thick coronal reformatted images.

### Imaging Interpretation

Three radiologists independently reviewed the sequences on a picture archiving and communication system (Visage Imaging, San Diego, CA). The radiologists, who are board certified and specialize in abdominal imaging with 3–19 years of academic experience, independently reviewed the MRE and CTE images to confirm the presence or absence of an internal fistula. The radiologists also reported imaging findings on a quantitative scoring sheet with room for subjective comments. The score sheet included assessment of radiographic evidence of intestinal inflammation, the presence of a fixed stricture, and whether or not these abnormalities were localized to the same diseased location as the fistula in question. The 3 radiologists agreed on the fistula status of 167 out of 199 cases leading to an 84% concordance rate. Differences were resolved by consensus. Radiologists were blinded to all corresponding patient data.

### Statistical Analysis

Comparison of variables between cases and controls was performed using simple logistic regression, with each predictor variable as the sole covariate and with “fistula” as the response. Multivariable logistic regression modeling was performed using clinical and radiologic variables as covariates. Variables were selected by constructing a causal directed acyclic graph (DAG) and determining the minimal adjustment set for measuring the effect of fistula presence. Kaplan–Meier function was used to estimate undesired time-free survival among different study groups. Data were analyzed using R (4.1.0).^11^

## Results

### Clinical Characteristics

The characteristics of cases and controls are shown in Table 1. The cases and controls were well matched for age, race, smoking status, body mass index, and years of disease. There was a trend toward increased percentage of female patients in the control group (female patients 50.7% vs 64.7%; *P* = .06). Not surprisingly, the cases were more likely to have a history of CD-related surgery (47.6% vs 30.2%; *P* = .02) and more likely to have a history of a prior intra-abdominal abscess (34.9% vs 5.9%; *P* < .001) with a higher proportion of abscesses within 1 year before the index scan in the cases (19.1% vs 2.2%; *P* < .001).

Regarding baseline laboratory work, cases had higher mean baseline C-reactive protein (52.1 ± 66.4 vs 13.3 ± 23.9 mg/L; *P* < .001), higher erythrocyte sedimentation rate (43.6 ± 35.3 vs 27.6 ± 22.8 mm/h; *P* = .005), lower hemoglobin (11.2 ± 2.1 vs 12.5 ± 1.9 g/dL; *P* = .003), and lower albumin (3.6 ± 0.7 vs 4.0 ± 0.6 g/dL; *P* < .001). Baseline HBI was comparable in cases and controls (6.8 ± 4.4 vs 6.72 ± 5.5; *P* = .9).

### Imaging

The index scan of cases was more likely to demonstrate radiographic evidence of inflammation (95.2% vs 39.7%; *P* < .001) and stricture (27.0% vs 7.35%; *P* = < .001) than controls.

### Follow-up Period

Average length of follow-up in case and control groups was comparable (24.3 ± 2.88 vs 27.0 ± 2.22 months; *P* = .40). The HBI was also comparable in both groups at the end of follow-up period (6.3 ± 4.8 vs 6.3 ± 5.1; *P* = .98).

### Medications

Data on different classes of medications including 5-aminosalicylic acid (eg sulfasalazine, Asacol, Pentasa, Rowasa), immunomodulators (azathioprine, methotrexate, 6-mercaptopurine, tacrolimus), biologics (infliximab, adalimumab, vedolizumab, ustekinumab, certolizumab pegol, and natalizumab), and Janus Kinase inhibitor (tofacitinib) were collected. Baseline medications, medications 6 months after the index scan, and at the end of the follow-up period were analyzed. There was no statistical significance between the groups in their medication class use initially or at 6 months of follow-up. Biologic use increased over time consistent with practice trends. Six months after the index scan, there was a 7.94% increase in biologic use in the fistula group compared with 11.72% in the controls (*P* = .11) and at the end of the follow-up period there were 26.98% and 27.94% additional biologic users in the fistula and control groups (*P* = .74), respectively. There was a trend toward higher rates of immunomodulatory use in the fistula group at the end of the follow-up period (49.2% vs 36.0%; *P* = .08). Although multiple changes made within the biologic class during the study period (ex, adalimumab to Ustekinumab), these changes were not statistically significant between both study groups (Table 2).

**Table 2:** Comparison Between Medication Use at Initial Visit and Follow-up Visit in Patients With Fistula on Imaging and Controls

Medication	Population (n = 199)	Fistula cases (n = 63)	No fistula cases (n = 136)	*P* value (cases vs controls)
Baseline medications				
Steroid within 2 wk	45 (22.6%)	17 (27%)	28 (20.6%)	.32
Mesalamine	32 (16.1%)	6 (9.5%)	26 (19.1%)	.07
Immunomodulators	53 (26.6%)	21 (33.3%)	32 (23.5%)	.15
Biologics within 8 wk	81 (40.7%)	30 (47.6%)	51 (37.5%)	.18
Jak inhibitors	5 (2.5%)	1 (1.6%)	4 (2.9%)	.55
Change in medications within 6 mo				
Steroid within 2 wk	24 (12.1%)	11 (17.5%)	13 (9.6%)	.12
Mesalamine	22 (11.1%)	5 (8%)	17 (12.5%)	.33
Immunomodulators	52 (27.1%)	17 (27%)	37 (27.2%)	.97
Biologics within 8 wk	102 (51.3%)	35 (55.6%)	67 (42.3%)	.41
Change within biologics	14 (7.04%)	9 (12.3%)	5 (4%)	.053
Jak inhibitors	1 (0.5%)	0 (0%)	1 (0.7%)	.38
End of follow-up meds				
Steroid within 2 wk	20 (10.1%)	7 (11.1%)	13 (9.6%)	.74
Mesalamine	14 (7%)	2 (3.2%)	12 (8.8%)	.12
Immunomodulators	80 (40.2%)	31 (49.2%)	49 (36%)	.08
Biologics within 8 wk	136 (36.3%)	47 (74.6%)	89 (65.4%)	.19
Change within biologics	32 (16.1%)	12 (16.4%)	20 (15.9%)	.99
Jak inhibitors	6 (3%)	0 (0%)	6 (4.4%)	.03

### Health Care Utilization

Overall, there was increased health care utilization by the fistula group. The average number of imaging studies (computed tomography or magnetic resonance imagingabdomen) during follow-up period was 4.76 ± 4.62 vs 3.04 ± 2; *P*= < .001. The percentage of patients who underwent abdominal surgery in the fistula group was 44.4% vs 24.3% of controls, *P* < .001. More patients from the fistula group requiring an ostomy (15.9% vs 9.6%; *P* = .021). There was a higher number of hospitalizations related to CD in the cases (68.25% vs 47.1%; *P* = .005). There was a greater incidence of intra-abdominal abscess formation in the fistula group (19.1% vs 3.7%, *P* < .001) with average time to abscess being 12.59 months after index scan. The average number of clinic visits during follow-up were comparable between groups. Interestingly, there was slightly greater number of endoscopic procedures performed on the control group (1.65 ± 1.75 vs 1.02 ± 1.22; *P* = .01) compared to cases.

### Multivariate Analysis Findings

Multivariable analysis was performed on the population using the subject variables listed in Table 1. To select variables for logistic regression, a DAG containing each variable in Table 1 was constructed for each response. In the constructed DAGs, a directed arc from one node (the parent variable) to another node (the child variable) indicates that the parent variable potentially has a causal influence on the child variable. For instance, because a patient’s provider may increase the patient’s medications in response to observed inflammation on the patient’s index scan, the constructed DAGs included a directed arc from the variable representing inflammation at the index scan to each variable representing a patient’s medication. To construct each DAG, all arcs representing plausible relations between variables were included, and arcs representing impossible relations—such as those where the parent variable was observed chronologically after the child variable—were excluded.

For each response, variables for logistic regression were selected by calculating the minimal adjustment set determined by the corresponding DAG for measuring the total effect of fistula status on the response (ie hospitalization, abscess, obstruction, surgery). A “minimal adjustment set” represents the smallest set of variables which are needed to render an unconfounded (independent) estimate of the effect of a variable (in this case, fistula status) on the response.^12^

Logistic regression with the minimal adjustment set as covariates for predicting hospitalization, intra-abdominal abscess development, obstruction, and abdominal surgery are shown in Table 3, Table 4, Table 5, Table 6, respectively. After multivariable analysis, patients with a fistula were more likely to be hospitalized (odds ratio [OR] 2.87, *P* = .016) than patients without fistula. Patients with fistula were more likely to develop an intra-abdominal abscess (OR 7.86, *P* = .032) and more likely to require surgery (OR 4.22, *P* = .002) compared to patients without fistula. Consistent with prior studies,^1^^,^^2^ logistic regression also revealed a relationship between having a stricture and development of an obstruction (OR 5.43, *P* = .0022).

**Table 3:** Logistic Regression Model Minimal Adjustment Set as Determined by DAGs Using the Subject Variables

Predictor	Coefficient estimate	Odds ratio	Coefficient standard error	Coefficient Z value	Coefficient *P* value
Intercept	0.86	2.35	0.82	1.04	.30
Age	−0.01	0.99	0.01	−0.74	.46
Gender: Male	0.03	1.03	0.36	0.09	.93
Race: Black	0.70	2.02	0.47	1.49	.14
Race: Other	0.61	1.84	1.01	0.60	.55
Smoker: Former smoker	−0.06	0.94	0.48	−0.12	.91
Smoker: Smoker	0.21	1.23	0.41	0.51	.61
BMI	−0.04	0.96	0.03	−1.39	.16
Fistula at imaging	**1.05**	**2.87**	**0.44**	**2.41**	**.02**
Stricture at imaging	0.76	2.14	0.53	1.44	.15
Inflammation at imaging	**−0.79**	**0.45**	**0.40**	**−1.97**	**.05**
Abdominal pain at imaging	**0.98**	**2.65**	**0.36**	**2.73**	**.01**
Intra-abdominal abscess history	−0.62	0.54	0.65	−0.96	.33
Abscess within 1 y prior	0.70	2.02	0.86	0.82	.41
Steroids within 2 wk prior imaging	**0.82**	**2.28**	**0.41**	**1.98**	**.05**
Mesalamine within 2 wk prior imaging	−1.15	0.32	0.47	−2.46	.01
Immunomodulators within 2 wk prior imaging	−0.46	0.63	0.39	−1.16	.24
Biologics within 8 wk prior imaging	−0.38	0.68	0.35	−1.10	.27

Summary for predicting patient hospitalization.

Bold values indicate statistically significant findings.

BMI, body mass index.

**Table 4:** Logistic Regression Model Minimal Adjustment Set as Determined by DAGs Using the Subject Variables

Predictor	Coefficient estimate	Odds ratio	Coefficient standard error	Coefficient Z value	Coefficient *P* value
Intercept	−4.03	0.02	2.09	−1.93	.05
Age	0.02	1.02	0.02	1.22	.22
Gender: Male	0.30	1.35	0.67	0.45	.65
Race: Black	−0.29	0.75	0.97	−0.30	.76
Race: Other	−1.34	0.26	1.98	−0.68	.50
Smoker: Former smoker	0.62	1.82	0.94	0.66	.51
Smoker: Smoker	−0.98	0.37	0.88	−1.12	.26
BMI	−0.10	0.91	0.07	−1.35	.18
Fistula at imaging	**2.06**	**7.87**	**0.96**	**2.14**	**.03**
Stricture at imaging	−3.02	0.05	1.68	−1.80	.07
Inflammation at imaging	0.03	1.03	0.98	0.03	.97
Abdominal pain at imaging	1.25	3.49	0.81	1.54	.12
Intra-abdominal abscess history	0.81	2.25	1.14	0.71	.48
Abscess within 1 y prior	1.47	4.33	1.23	1.20	.23
Steroids within 2 wk prior imaging	1.11	3.02	0.69	1.61	.11
Mesalamine within 2 wk prior imaging	0.40	1.50	1.02	0.40	.69
Immunomodulators within 2 wk prior imaging	0.57	1.76	0.81	0.70	.48
Biologics within 8 wk prior imaging	0.40	1.50	0.68	0.59	.55

Summary for predicting abscess formation.

Bold values indicate statistically significant findings.

BMI, body mass index.

**Table 5:** Logistic Regression Model Minimal Adjustment Set as Determined by DAGs Using the Subject Variables

Predictor	Coefficient estimate	Odds ratio	Coefficient standard error	Coefficient Z value	Coefficient *P* value
Intercept	−1.20	0.30	1.09	−1.10	.27
Age	0.01	1.01	0.01	0.69	.49
Gender: Male	0.58	1.79	0.44	1.33	.18
Race: Black	−0.14	0.87	0.65	−0.21	.83
Race: Other	−0.24	0.79	1.19	−0.20	.84
Smoker: Former smoker	−0.24	0.79	0.70	−0.34	.73
Smoker: Smoker	−0.12	0.89	0.50	−0.24	.81
BMI	−0.07	0.94	0.04	−1.59	.11
Fistula at imaging	−0.50	0.61	0.53	−0.95	.34
Stricture at imaging	**1.69**	**5.43**	**0.55**	**3.06**	**.00**
Inflammation at imaging	0.63	1.88	0.53	1.19	.24
Abdominal pain at imaging	0.23	1.26	0.46	0.50	.62
Intra-abdominal abscess history	−0.91	0.40	0.94	−0.96	.34
Abscess within 1 y prior	1.14	3.12	1.15	0.99	.32
Steroids within 2 wk prior imaging	0.87	2.40	0.46	1.90	.06
Mesalamine within 2 wk prior imaging	−0.33	0.72	0.62	−0.53	.59
Immunomodulators within 2 wk prior imaging	−0.14	0.87	0.48	−0.29	.77
Biologics within 8 wk prior imaging	−0.10	0.91	0.43	−0.22	.82

Summary for predicting obstruction.

Bold values indicate statistically significant findings.

BMI, body mass index.

**Table 6:** Logistic Regression Model Minimal Adjustment Set as Determined by DAGs Using the Subject Variables

Predictor	Coefficient estimate	Odds ratio	Coefficient standard error	Coefficient Z value	Coefficient *P* value
Intercept	−1.85	0.16	0.96	−1.92	.05
Age	−0.02	0.98	0.01	−1.59	.11
Gender: Male	0.43	1.53	0.38	1.10	.27
Race: Black	−0.48	0.62	0.54	−0.88	.38
Race: Other	0.94	2.55	0.88	1.07	.29
Smoker: Former smoker	0.08	1.09	0.59	0.14	.89
Smoker: Smoker	0.03	1.03	0.45	0.06	.95
BMI	0.01	1.01	0.03	0.16	.87
Fistula at imaging	**1.44**	**4.23**	**0.47**	**3.07**	**.00**
Stricture at imaging	0.83	2.29	0.54	1.55	.12
Inflammation at imaging	0.04	1.04	0.47	0.09	.93
Abdominal pain at imaging	0.20	1.22	0.41	0.48	.63
Intra-abdominal abscess history	−0.38	0.69	0.72	−0.53	.60
Abscess within 1 y prior	1.56	4.74	0.92	1.69	.09
Steroids within 2 wk prior imaging	**1.04**	**2.85**	**0.43**	**2.42**	**.02**
Mesalamine within 2 wk prior imaging	**1.07**	**2.90**	**0.50**	**2.14**	**.03**
Immunomodulators within 2 wk prior imaging	−0.52	0.60	0.44	−1.17	.24
Biologics within 8 wk prior imaging	0.43	1.53	0.39	1.09	.27

Summary for abdominal surgery.

Bold values indicate statistically significant findings.

BMI, body mass index.

To better control for cofounders, a subgroup analysis was performed after excluding patients with history of Crohn’s-related surgery within 6 months of the index scan; 5 patients were in the fistula group and 4 patients were in the control group. In this subgroup, patients with fistula remained more likely to require surgery (OR 4.43, *P* = .0021), require hospitalization (OR 3.00, *P* = .014), or develop an intra-abdomen abscess (OR 10.14, *P* = .027).

To ensure abscess development was not due to a recent prior abscess, a subgroup was constructed by excluding patients with an abdominal abscess within 1 year before the index scan, and a logistic regression for predicting abscess development was performed. In this model, the effect of fistula presence remained significant (OR 5.2033, *P* = .01178).

### Survival Analysis

A survival analysis was performed to assess the time from initial imaging study to development of undesired outcome (need for abdominal surgery, development of obstruction, ostomy creation and abscess formation). Results showed that patients with fistula on imaging required sooner undesired outcome compared with patients without fistula (*P* < .001) Figure.

**Figure:**
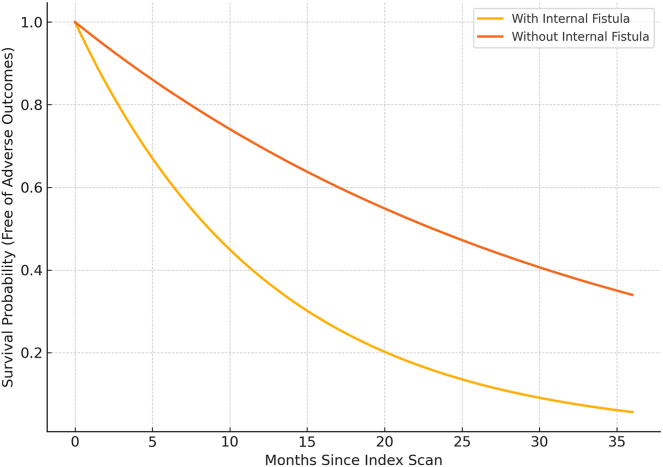
Survival probability for adverse outcomes in CD. Kaplan–Meier survival analysis comparing patients with and without internal fistulas identified on cross-sectional imaging. Patients with imaging findings of fistula experienced statistically significant earlier onset of severe outcomes—including abdominal surgery, bowel obstruction, ostomy creation, and intra-abdominal abscess formation—compared to those without fistulas (*P* < .01).

## Discussion

Patients with CD have multiple cross-sectional imaging studies during the course of their disease. CSI is considered the standard of care for assessing small intestinal inflammation and for identifying complications of the disease.^4^ CSI can be used to predict important disease outcomes including obstructions and response to therapy.^1^^,^^2^ Our study adds to the predictive abilities of routine CSI by demonstrating that an internal fistula identified on CSI predicts important future complications including internal abscess formation and the need for surgery. This single radiographic finding is important regardless of patient symptoms.

The probability of finding an internal fistula on CSI was 31.6% in our study. In other studies, internal fistulas are found on approximately 15% of scans in CD patients.^6^ The difference likely reflects several factors. First, our tertiary care center’s patient population at the University of Florida Health Shands Hospital is a Medicare Disproportionate Share Hospital serving a large number of Medicaid and uninsured individuals from north central Florida and southern Georgia that may present with more advanced disease. Further, MRE is the preferred modality for imaging patients with CD at our center due to less radiation and high sensitivity in identifying internal fistula which is comparable to CTE.^13^^,^^14^ Finally, all of the CSI in our study were read by experienced abdominal radiologists for the purposes of this study who may have been more focused on the fistula findings.

The group of patients with internal fistulas noted on index imaging had more active disease at the time of the index scan compared to controls based on history, baseline lab work, and inflammation observed on imaging. This is not surprising since patients with penetrating complications like fistulas have an overall more complicated disease course.^3^^,^^6^^,^^8^ With this in mind, medication use, particularly biologic use, would be expected to be higher in the patients with internal fistulas. There was no difference in biologic use between the fistula and control groups. A similar increase in biologic use was noted in both groups over time consistent with changing patterns of medication use. There was no change in biologic use in the fistula group within 6 months after the fistula was noted on cross-sectional imaging. This is consistent with the idea that the finding of an internal fistula is considered incidental and does not typically trigger intensification of therapy.

Cases had increased health care utilization during follow-up period compared with controls. This is consistent with their more complicated disease course. Cases had increased CD-related hospitalizations and increased number of CSI studies. They were more likely to require CD-related surgery and more likely to receive an ostomy. Interestingly, outpatient visits were comparable between groups and procedures, including colonoscopies, were slightly greater in the control group. These latter findings are unexpected in that patients with fistulas have more complicated disease courses and would be expected to result in higher rates of outpatient visits indicating closer follow-up.

Our study demonstrated that patients with internal fistulas were more likely to develop an intra-abdominal abscess. Intra-abdominal abscesses are complications that are extremely challenging for patients and physicians. In the setting of an intra-abdominal abscess, surgery is more complicated and when performed, is more likely to result in the need for an ostomy. Medically, biologics are contraindicated in the presence of an undrained abscess. While not well studied, good outcomes can be achieved with percutaneous drainage and antibiotics until imaging suggests the abscess is well drained, then biologics used for weeks to months before surgical resection. In most cases, oral nutrition is maintained and TPN can be avoided though this depends on the nutritional status of the patient.^15^^,^^16^ Our finding that an internal fistula is the only clinical variable predictive of the development of an abscess highlights the power of CSI. The finding of an internal fistula on CSI should alert the physician to the possibility of a future abscess and patients should be aware of the complicated course ahead if an abscess develops.

Internal fistulas are often considered incidental findings on CSI. Surgeons are reluctant to operate if the patient has no symptoms that could be improved with surgical intervention. Similarly, gastroenterologists and patients may be reluctant to use biologics or other immunosuppressive therapies in an asymptomatic patient. Data suggest that biologic therapy is able to “heal” the internal fistula in up to 40% of cases.^17^ In our study, the difference in frequency of reported abdominal pain between the groups was not statistically significant. Our data suggest that the finding of an internal fistula is not incidental but rather forewarns of serious adverse outcomes like an abscess and the need for surgery. Therefore, biologic use is warranted independent of the patient’s symptoms.^6^

CSI is the modality of choice for assessing small intestinal CD severity affecting the small intestine. Studies in CD have used a robust scoring system to assess disease severity: magnetic resonance index of activity.^18^^,^^19^ Using CSI to provide prognostic information about the natural history of disease processes and predict disease outcomes is commonly used in oncology and nonmalignant diseases.^13^^,^^14^^,^^20^^,^^21^ In CD, a study from our group demonstrated that a fixed stricture on routine CSI predicted disease-related complications and adverse outcomes.^1^ In that study, a patient with a fixed stricture in the setting of radiographic evidence of active inflammation was 15 times more likely to develop a complication such as bowel obstruction, perforation, internal fistula or abscess in the subsequent 2 years.^1^ In our new study, 26.9% of patients had a fixed stricture associated with the internal fistula compared with 7.3% of controls. A fixed stricture on CSI predicted the development of obstruction and patients with a fixed stricture on index imaging were 5.73 times more likely to develop a subsequent obstruction. However, a fixed stricture did not predict the development of an abscess.

Our study has several limitations. It is retrospective in design with limitations in data collection and is not able to account for changes in practice patterns over time. Our patient population with its high rate of advanced CD may amplify the rate of complications and limit the ability to generalize our findings. Our study highlights the importance of the finding of an internal fistula but does not allow determination of best management practices. Our findings set the stage for a multicenter trial to confirm our findings in a larger, diverse population and a subsequent prospective, randomized trial of medical vs surgical therapy.

## Conclusion

We have shown for the first time that the presence of an internal fistula on cross-sectional imaging predicts the development of future severe complications regardless of patient symptoms. An internal fistula is the most important risk factor for the development of an intra-abdominal abscess in a patient with CD. The presence of an internal fistula should not be considered an incidental finding but rather an important piece of prognostic information that will help gastroenterologists and surgeons better inform their patients and provide a more accurate risk/benefit analysis for potent immunosuppressive therapy and surgery.
