# Supplementary material for: Internal Fistulas Discovered on Cross-Sectional Imaging Predict Future Intra-Abdominal Abscesses
Source: Gastro Hep Adv. 2025 Apr 25;4(8):100684. doi: 10.1016/j.gastha.2025.100684 (PMC12209915; doi:10.1016/j.gastha.2025.100684)
Supplement: Supplemental File [file mmc1.docx]

MRN ____________ Case Number_____________ Date of study ______________

Today’s date: _______________ Test Set Y/N

Type of study (circle answer): 1= CTE 2 = MRE 3= CT Abd 4= other

Name of Radiologist: a) XXXXX b) XXXXX c) XXXXX

| **Fistula present**  Yes/No | Yes (1) No (0) |
| --- | --- |
| **Type of fistula** 0=none 1=sinus tract 2=simple (bowel, bladder etc.) 3=complex (multiple with SB, LB, bladder, other) | 0 1 2 3 |
| **Location of Fistula origin**  Jejunum (prox, med, distal)  Ileum (prox, mid, terminal) other (describe) |  |
| **Location of Fistula destination**  Small bowel, cecum, sigmoid, rectum, bladder, complex, other (describe) |  |
| **Slice** (Representative) |  |
| **Presence of Perienteric Fluid** 0= None  1= edema 2= inflammatory mass 3= abscess | 0 1 2 3 |
| Distance of Surgical margin to fistula (cm w/ one decimal) |  |
| **Location of Diseased Bowel Segment** (nearest to origin of fistula) May be same as the Location of Fistula Origin |  |
| **Inflammation of the Diseased Segment** (Y/N) | Yes (1) No (0) |
| **Length** (cm w/ one decimal) |  |
| **Wall Thickening**  (y/n) Mild= 3≤5 mm, Moderate= 5-9 mm, Severe ≥10 mm | Yes (1) No (0) 3-5 mm 6-9 mm ≥ 10 mm |
| **Enhancement** 1=Yes Segmental mural hyperenhancement 0=no/none | Yes (1) No (0) |
| **Ulceration**  (Y / N) | Yes (1) No (0) |
| **Intramural Edema (T2 fat sat)**  (Y / N) | Yes (1) No (0) |
| **Stricture of the Diseased Segment**  (Y / N) Yes= Upstream dil >3cm | Yes (1) No (0) |
| **Diameter of dilated bowel** (cm w/ one decimal) |  |
| **Minimal Lumen Diameter** (mm) |  |
| **Comments** |  |
